# Development and validation of a novel lysosome-related LncRNA signature for predicting prognosis and the immune landscape features in colon cancer

**DOI:** 10.1038/s41598-023-51126-9

**Published:** 2024-01-05

**Authors:** Fengming Li, Wenyi Wang, Guanbiao Lai, Shiqian Lan, Liyan Lv, Shengjie Wang, Xinli Liu, Juqin Zheng

**Affiliations:** 1 Center of Digestive Endoscopy, Longyan First Affiliated Hospital of Fujian Medical University, Longyan, China; 2 Department of Medical Oncology, Xiamen Key Laboratory of Antitumor Drug Transformation Research, The First Affiliated Hospital of Xiamen University, School of Medicine, Xiamen, China; 3 Department of Thyroid and Breast Surgery, Xiamen Humanity Hospital Fujian Medical University, Xiamen, Fujian China; 4 Department of Medical Oncology, Xiang’an Hospital of Xiamen University, School of Medicine, Xiamen University, Xiamen, China

**Keywords:** Prognostic markers, Gastrointestinal cancer, Tumour biomarkers, Tumour immunology, Cancer genomics

## Abstract

Lysosomes are essential components for managing tumor microenvironment and regulating tumor growth. Moreover, recent studies have also demonstrated that long non-coding RNAs could be used as a clinical biomarker for diagnosis and treatment of colorectal cancer. However, the influence of lysosome-related lncRNA (LRLs) on the progression of colon cancer is still unclear. This study aimed to identify a prognostic LRL signature in colon cancer and elucidated potential biological function. Herein, 10 differential expressed lysosome-related genes were obtained by the TCGA database and ultimately 4 prognostic LRLs for conducting a risk model were identified by the co-expression, univariate cox, least absolute shrinkage and selection operator analyses. Kaplan–Meier analysis, principal-component analysis, functional enrichment annotation, and nomogram were used to verify the risk model. Besides, the association between the prognostic model and immune infiltration, chemotherapeutic drugs sensitivity were also discussed in this study. This risk model based on the LRLs may be promising for potential clinical prognosis and immunotherapeutic responses related indicator in colon cancer patients.

## Introduction

Colon cancer, one of the most common digestive tract tumors, has been widely appreciated as the third most prevalent cancer and the second most likely cause of death based on data published by the World Health Organization (WHO)^1–3^. Currently, the prognosis of colorectal cancer is closely related to the tumor stage, the five-year survival rate of stage I colorectal cancer is 90%, while the five-year survival rate of stage IV colorectal cancer with distant metastasis is only 14%^4^. Patients with early-stage colorectal cancer generally have no obvious clinical symptoms, and screening and diagnostic tools are very limited, thereby resulting in the continuous increase of the incidence of colon cancer^5,6^. Consequently, it is urgent and necessary to explore potential biomarkers to prevent and treat colon cancer.

Lysosomes are important regulators of homeostasis in cells and organisms, mediating energy metabolism, cell proliferation and differentiation, immunity, and cell death^7–9^. Recent studies have demonstrated that the dynamics of lysosomes played an important role in the development and progression of malignant tumors^10,11^. For example, lysosome associated membrane protein family member 1 (LAMP1) has been reported to catalyze the formation of CagA-degrading autolysosomes, further increasing risk of gastric carcinogenesis^12^. Knockout of the small non-coding VTRNA1-1 in human hepatocellular carcinoma cells could reduce nuclear localization of TFEB (Central regulator of the autophagolysosomal signaling pathway) and lysosomal compartment dysfunction, thereby inhibition of tumor proliferation^13^. Besides, Nie et al. reported that lysosomal mediated cytotoxic autophagy promotes colon cancer cells apoptosis through mTOR-TFEB signaling^14^. Therefore, searching for new lysosomal targeting molecules may be a promising strategy for cancer therapy.

Long non-coding RNAs (lncRNAs) are a class of non-coding RNAs containing more than 200 nucleotides, most of which have no protein-coding potential, responsible for epigenetic regulation, cell cycle control and DNA damage, regulation of microRNA, participation in signal transduction pathways, mediating hormone-induced cancer, etc.^15–18^. Numerous evidence has demonstrated that the dysfunction of lncRNAs was closely related to the occurrence, development, recurrence, metastasis and chemotherapy resistance of tumors^19–21^. For example, lncRNA LINC00662 overexpression could accelerate the growth and metastasis of colon cancer by activating ERK signaling pathway, and m^6^A-modified lncRNA GAS5 has also been shown to involve in the hippo pathway, and subsequently regulate the progression of colorectal cancer^22,23^. Additionally, a recent study reported that ferroptosis-related lncRNA signatures as a novel prognostic model for predicting the clinical outcomes in colon cancer patients through extracting TCGA and FerrDb databases**,** and validated knockdown of AP003555.1 and AC000584.1 could decrease the colon cancer cell proliferation ability in vitro^24^. However, the prospect of lysosome-lncRNA combinations in prognostic prediction for colon cancer patients remains to be elucidated.

In this study, we utilized the transcriptional data of colon cancer from TCGA database, identified ten differentially expressed lysosome-related genes and screened out four lysosome-related lncRNAs by co-expression analysis, then we further constructed a prognostic model and evaluated its association with clinical traits, immune infiltration, and chemotherapy drug sensitivity. Our results could provide valuable strategies and excavate lysosome related promising direction for colon cancer therapy.

## Materials and methods

### Data extraction

We downloaded the clinical, transcriptional, and mutation data of colon cancer from the TCGA database (https://portal.gdc.cancer.gov/). Using the following search strategy: (Primary Site IS colon AND Program Name IS TCGA AND Project Id IS TCGA-COAD AND Data Category IS Clinical AND Data Format IS bcr xml), (Primary Site IS colon AND Program Name IS TCGA AND Project Id IS TCGA-COAD AND Workflow Type IS STAR—Counts AND Data Category IS transcriptome profiling AND Data Type IS Gene Expression Quantification), and (Primary Site IS colon AND Program Name IS TCGA AND Project Id IS TCGA-COAD AND Data Category IS simple nucleotide variation AND Data Type IS Masked Somatic Mutation). We downloaded a total of 517 sample data, including 476 COAD samples and 41 normal tissues. The mRNA and lncRNA expression matrices from TCGA-COAD cohort were extracted via R package (Limma). For clinicopathological signatures, we recorded the survival status, survival time, age at diagnosis, gender, and clinical stage of each patient.

### Lysosome-related genes and lncRNA expression data extraction

**Figure 1:**
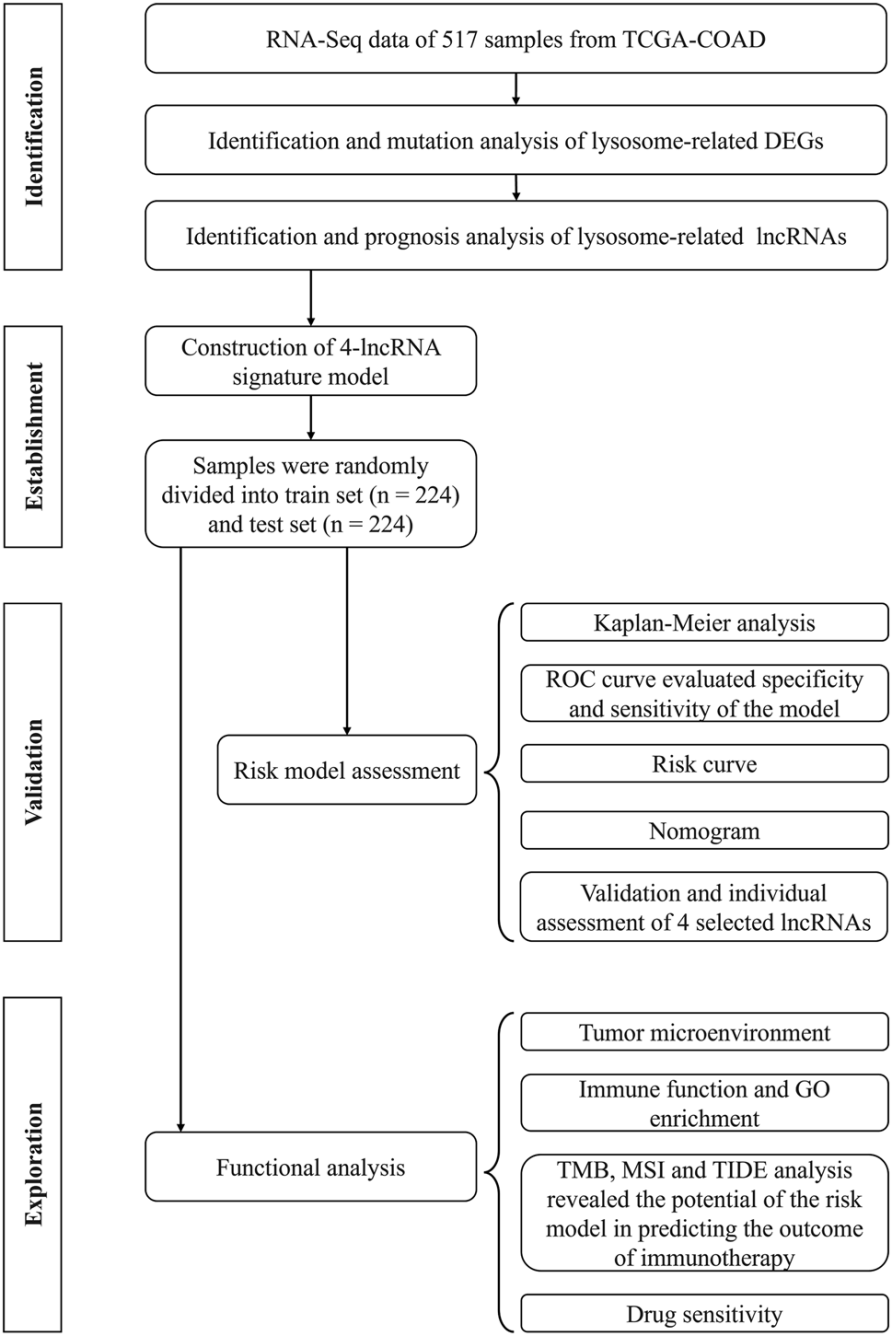
Workflow of this study.

Sixty-one Lysosomal-related genes were retrieved from the GSEA dataset (https://www.gseamsigdb.org/gsea/msigdb/human/geneset/LYSOSOME.htmland^25^, we further extracted the expression profile of lysosomal-related genes by R package (Limma). Ten differential expressed lysosomal-related genes were subsequently screened with the criteria of |log _2_ (fold change) |> 1 and a false discovery rate (FDR) < 0.05. Subsequently, spearman correlation coefficients were calculated based on LRGs and lncRNA expression profiles to identify LRLs (selection criterion: |R| > 0.4 and *p* < 0.05). The flow chart of overall procedures is shown in Fig. 1.

### Differential expression of lysosome-related lncRNAs

Differentiation expressions of lncRNAs were calculated via the R package (Limma) with the selection criterion of |log _2_ (fold change) |> 1 and a *p* value < 0.05^26^. Further analysis was conducted with univariate COX-regression analysis (*p* < 0.05) to screen differentially expressed lncRNAs related to the 5-year OS of COAD patients and visualized via forest plot. Heatmap and boxplot of the expression of ten prognostic lncRNAs between normal and tumor samples were visualized via the R package (heatmap, ggpubr)^27^.

### Establishment of a risk score model and validation of its prognostic value

We performed Lasso and multistate COX-regression analysis to establish the risk score model. After confirming the model, the identified lncRNAs corresponding multivariate COX-regression coefficient and gene expression level were used to calculate the risk score. Samples calculated the risk score according to the sum of the product of expression data and the corresponding coefficient of each lncRNA. We set the median risk score as the criteria for differentiating between the high- and low-risk groups. Samples were randomly sorted into the Train and Test groups to double-check the outcome by R package (caret). Further, the Kaplan–Meier survival curve and heatmap demonstrated the relevance of risk score and prognosis (survminer, heatmap).

$$Risk\;score = \sum\limits_{i = 1}^{n} {\left( {lncRNAexp_{i} \times coef_{i} } \right)}$$

### Nomogram

Prognostic nomogram that combined the risk scores and clinical risk factors were constructed by R package (rms) to provide a quantitative method for personalized survival prediction. Prognostic accuracy of the nomogram at 1,3,5-years was compared and revealed by calibration curves.

### Tumor microenvironment and immune infiltration analysis

Tumor microenvironment (TME) analysis was performed via the R package (ESTIMATE), and followed by coming out with stroma score (representing the proportion of stroma cells), immune score (denoting the balance of immune cells), and estimate score (meaning the combination of stroma score and immune score). We retrieved the immune infiltration matrix from CIBERSORT and examined the differential distribution of ssGSEA based on twenty-two immune cells between high- and low-risk groupsl, and visualized by R package (ggpubr)^28^.

### Tumor mutational burden (TMB) and microsatellite instability (MSI) analysis

The somatic mutations of the COAD patients were devided into high- and low-risk groups and analyzed via R package (maftools)^29^. Additionally, we calculated the correlation between the risk score and MSI score via R package (plyr) and visualized via R package (ggpubr). MSI data was downloaded by using the R package (cBioPortalData) from cBioPortal database (http://www.cbioportal.org/^30^.

### Function annotation and drug susceptibility analysis

We conducted Gene Ontology (GO) analysis on the differentially expressed lncRNA genes between the high-risk and low-risk groups using R package (clusterProfiler), and *p* value < 0.05 was considered statistically significant^31^. Besides, the “oncoPredict” program was also employed to evaluate the IC50 of chemotherapy drugs, aiming for better predicting the therapeutic effects between high- and low- risk groups of LRLs^32^.

### Cell culture and quantitative real-time polymerase chain reaction

Normal colonic epithelial cells (HCoEpiC) and three colon cancer cells (SW480, SW620 and HCT116) were obtained from the American Type Cultural Collection (ATCC) and cultured based on the manufacture instructions, and the above cells used were not contaminated with mycoplasma. Until the cell density reaches 80%, the total RNA of each group could be collected and followed analyzed the target genes expression. The SPARKeasy cellular RNA extraction kit (AC0205, Shandong Sparkjade Biotechnology Co., Ltd.) and the Evo M-MLV RT Kit (AG11711, Accurate Biotechnology (Hunan) Co., Ltd. (Changsha, China)) were applied for the cDNA synthesis. The Hieff®qPCR SYBR Green Master Mix (11201ES08) was purchased from Yeasen Biotechnology (Shanghai) Co., Ltd. (Shanghai, China). The 2^-ΔΔCT^ formula was employed for measuring the relative expression value of target genes. The correlation analysis was conducted by one-way ANOVA test and *p < 0.05, **p < 0.01, ***p < 0.001. The primers used in this study were listed in Table S1.

### Statistical analysis

COX regression model was utilized to analyze the risk-associated lncRNAs involving the prognosis of patients; the hazard ratio (HR) and 95% confidence interval (95% CI) were calculated. *p* < 0.05 was considered statistically significant. The prognostic ability of the derived prognostic signatures for colon cancer paitient was assessed by receiver operating characteristic (ROC) curve analysis and visualized via R package (survivalROC), and higher the area under the curve (AUC) value demonstrated the higher sensitivity and specificity of the lysosome-related lncRNA-based risk model. PCA was performed via three parameters of all genes, LRLs, and LRLs that selected in the risk model. Additionally, we analyzed the risk score from the lysosome-related lncRNA-based model in age, gender, and clinical stage for predicting 5-year survival.

### Ethics approval and consent to participate

This experimental study does not involve animal or clinical studies and does not require submission for ethical review.

### Consent for publication

All authors gave permission for this study to be published.

## Results

### Screening of the differential expressed LRGs and description of the genetic mutations

**Figure 2:**
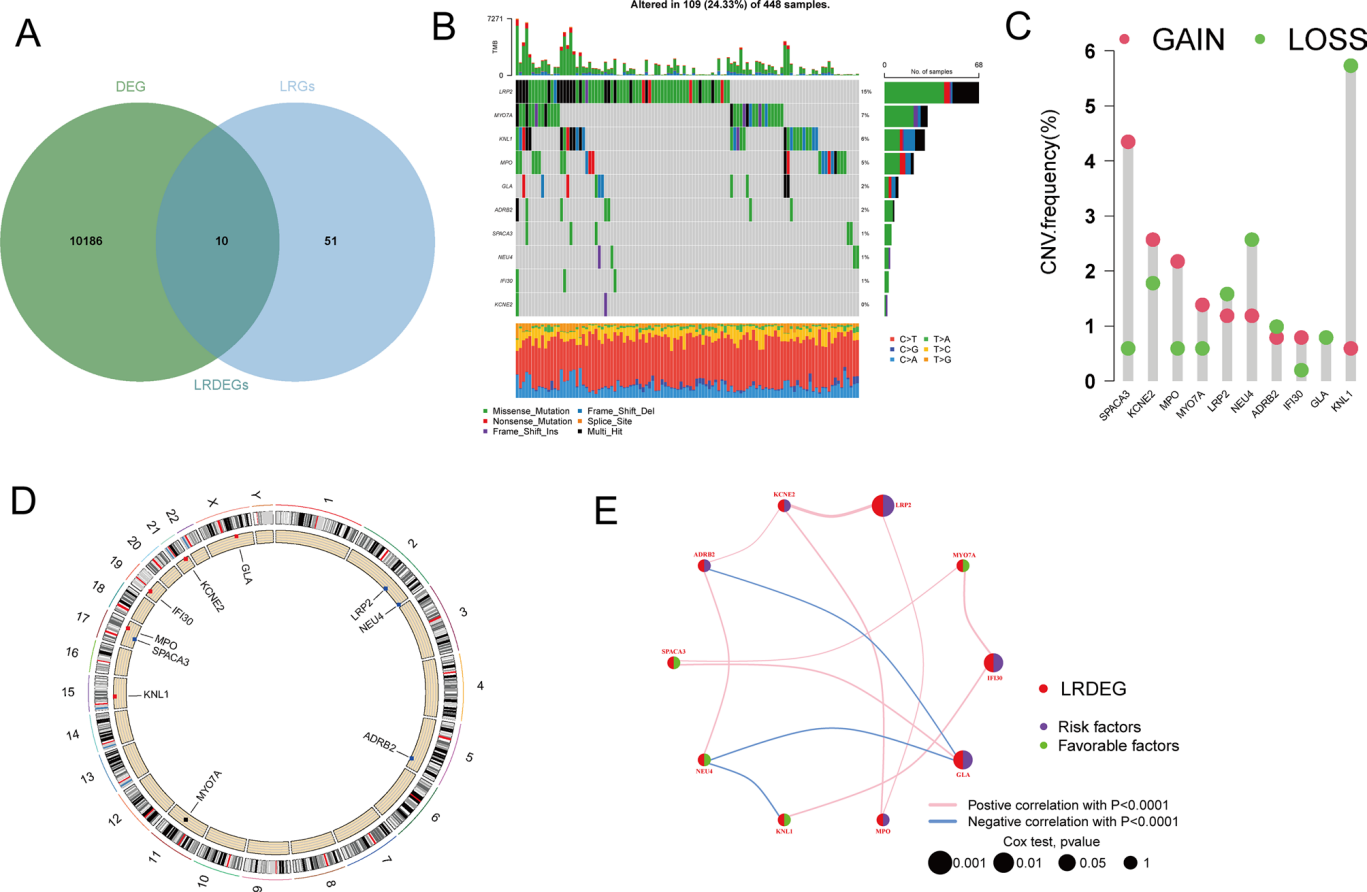
Screening and genetic mutation of LRDEGs. (**A**) intersection of tumor and non-tumor DEGs and LRGs. (**B**) Genetic variation in 10 LRDEGs. (**C**) Frequency of CNV in 10 LRDEGs. (**D**) LRDEGs distribution across 23 chromosomes. (**E**) Correlation network of 10 LRDEGs in the TCGA cohort.

Figure 1 shows the workflow process. We primarily retrieved 476 colon tumor and 41 normal samples from the TCGA database to filtrate out the optimal differential expressed genes (DEGs) with the criteria of the log FC > 1, *p* value < 0.05, and the result displayed 10,196 DEGs in the study. Subsequently, we downloaded 61 lysosome related genes (LRGs) from the GSEA dataset and intersected with the DEGs, ultimately, 10 LRDEGs in total were collected (Fig. 2A). Then, 10 LRDEGs in the colon cancer cohort were showed for the presence of CNVs and somatic mutations. As is illustrated in Fig. 2B, 109 (24.3%) samples occurred obvious mutations, and the most often mutated of the genes was LRP2 (15%), followed by the MYO7A (7%) and KNL1 (6%). Besides, the copy number variations of the above 10 LRDEGs were examined in Fig. 2C and the location of chromosome distribution of these genes were also displayed in Fig. 2D. Using a network format, the extensive interaction landscape, regulator interconnections and clinical outcome of 10 LRDEGS in colon cancer were comprehensively illustrated in this study (Fig. 2E).

### Identification of lysosome-related lncRNAs in patients with colon cancer

**Figure 3:**
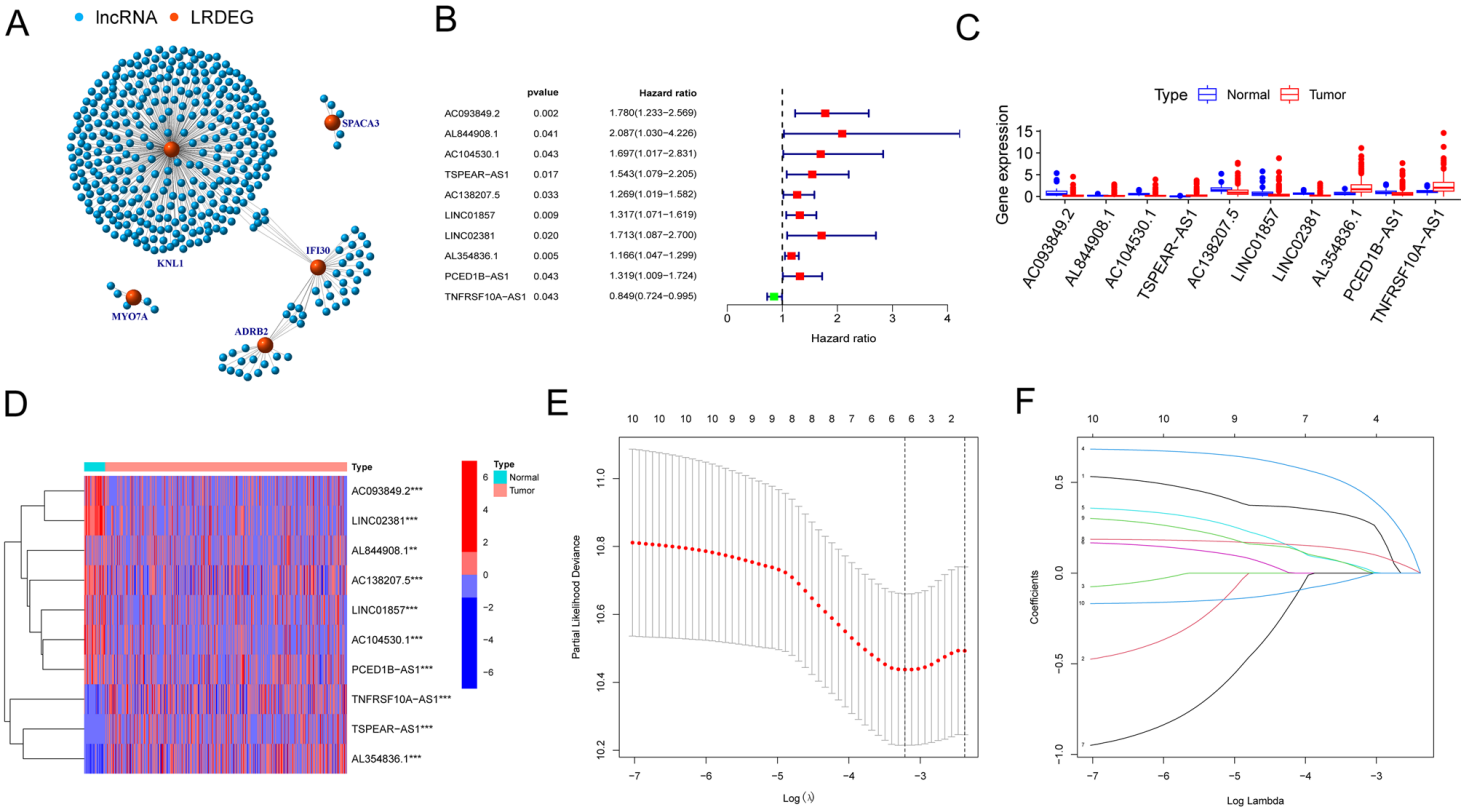
identification of lysosome related lncRNAs (LRLs) and constructing risk model for colon cancer patients based on LRLs. (**A**) A visual network diagram for 10 LRDEGs and their related lncRNAs. (**B**) Univariate Cox regression analysis revealing that association with the selected LRLs and clinical prognosis. (**C**, **D**) Boxplot and heatmap illustrating the expression patterns of 10 prognostic LRLs in colon cancer, respectively. (**E**) The tuning parameters (log λ) of OS-related proteins were selected to cross-verify the error curve. According to the minimal criterion and 1-se criterion, perpendicular imaginary lines were drawn at the optimal value. (**F**) The LASSO coefficient profile of 4 lncRNAs and perpendicular imaginary line were drawn at the value chosen by tenfold cross-validation.

Recent studies have demonstrated that the advancement of interaction prediction research in various fields of computational biology would provide valuable insights into genetic markers and lncRNAs related with malignancies^33–35^. Therefore, we conducted a coefficient expression analysis to establish mRNA-lncRNA network and screen the LRLs under the criteria of |R|> 0.4 and *p* < 0.05, and 388 lncRNAs were recognized as LRLs (Fig. 3A). To identify the prognostic value of these LRLs in colon cancer, we performed univariate COX regression analysis on differentially expressed lncRNAs, and the result showed that 10 lncRNAs were closely corrected with overall survival of patients with colon cancer (AC093849.2, AC093849.2, AC104530.1, TSPEAR-AS1, AC138207.5, LINC01857, LINC02381, AL354836.1, PCED1B-AS1 and TNFRSF10A-AS1, threshold: *p* < 0.05) (Fig. 3B). Additionally, differential expression of ten lysosome-related lncRNAs between tumor and adjacent tissue samples were analyzed via R package (Limma) and portrayed via boxplot and heatmap (ggpubr, heatmap) (Fig. 3C,D).

### Prognosis analysis of lysosome-related lncRNAs risk model

Based on the differential expression of lncRNAs, we identified four prognostic LRLs to establish a prognosis model for colon cancer patients by utilizing Lasso COX regression analyses (Fig. 3E,F). Subsequently, four lncRNAs (TNFRSF10A-AS1, AL354836.1, AC138207.5 and TSPEAR-AS1) were selected to build a risk model, and the coefficients of these lncRNAs were used to calculate the risk score. The risk score = TNFRSF10A-AS1*(0.164589534) + AL354836.1*0.167755259 + AC138207.5*0.315553626 + TSPEAR-AS1*0.653228553. With the median cutoff of risk score, patients are divided into high- and low-risk group.

**Figure 4:**
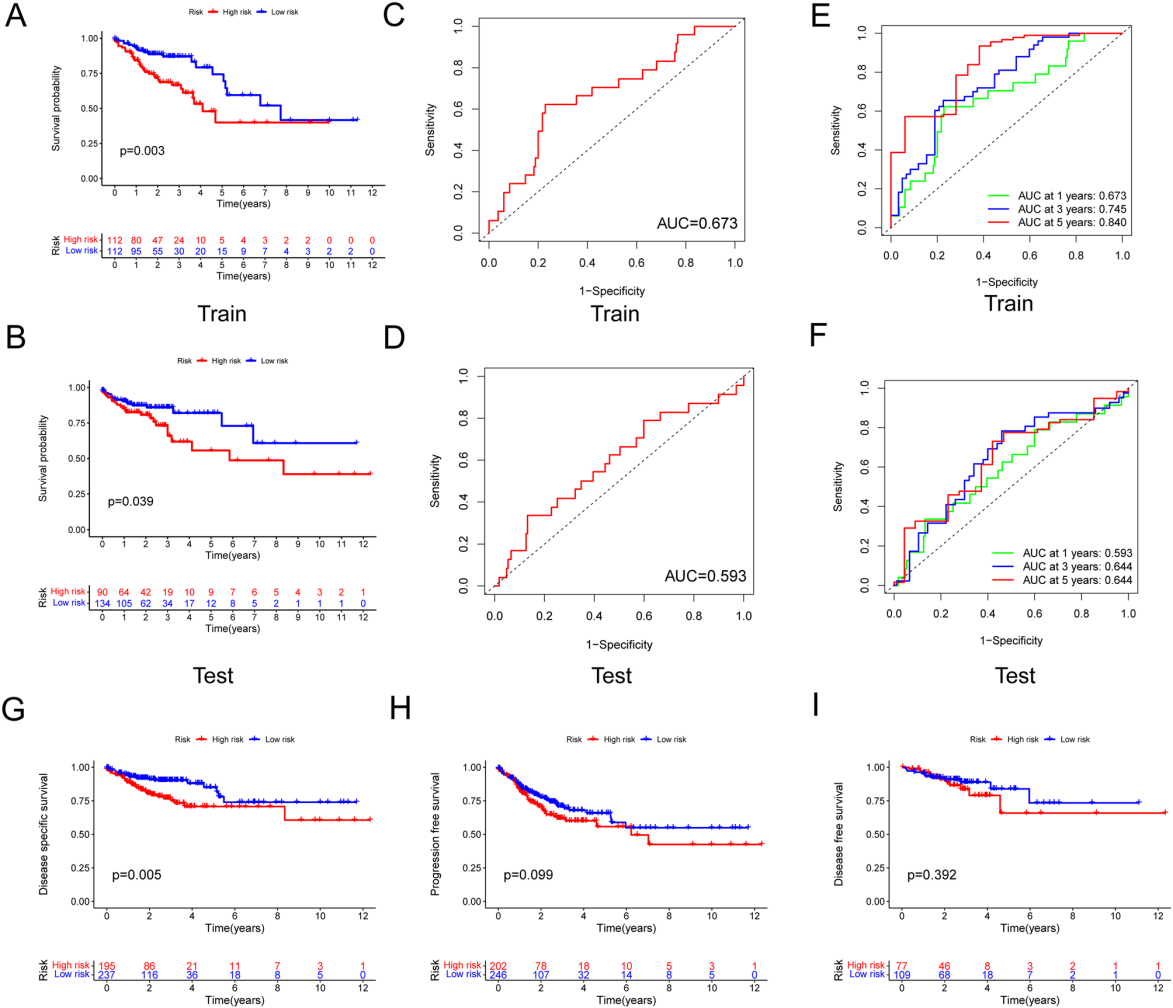
Prognostic value of the risk patterns of the 4 LRLs in the TCGA training set and test set. (**A**) Kaplan–Meier survival curve of patients with CRC in high- and low-risk groups train set. (**B**) The receiver operating characteristic (ROC) curve of four LRLs model in train set. (**C**) Kaplan–Meier survival curve of patients with CRC in high- and low-risk groups test set. (**D**) The receiver operating characteristic (ROC) curve of four LRLs model in test set. (**E**, **F**) Time-dependent ROC curve for predicting 1-, 3- and 5-year survival in the train and test group. (**G**, **I**) DSS, PFS and DFS of patients with CRC in high- and low- risk groups in train test. *p* < 0.05 was considered significant. DSS: Disease-specific survival; PFS: Progression-free survival; DFS: Disease-free survival.

To examine our risk model, we randomly separated patients into Train and Test groups equally in the high- and low-risk groups. The results implicated that in both the Train and the Test groups, the low-risk groups’ overall survival was significantly longer than that in the high-risk group (Fig. 4A,B). The ROC curve illustrated that the risk score has a solid predictive ability, with an AUC of 0.673 in the Train group (Fig. 4C) and 0.593 in the Test group (Fig. 4D). Totally, AUC values of 0.673, 0.745, and 0.840 were used to represent the 1-, 3-, and 5-year survival rates of LRLs_score in train set, and AUC values of 0.593, 0.644 and 0.644 were used in the test set, respectively (Fig. 4E,F). Furthermore, we also employed the disease-specific survival, progression-free survival and disease-free survival analysis for further evaluation the risk model prognostic, and found that the high- risk group had a worse disease-specific survival prognosis than the low- risk group (Fig. 4G-I). These analyses indicated that the risk model has relatively accurate furcating ability on clinical outcomes.

### The risk model and clinicopathological variables on the prognosis of colon cancer patients

**Figure 5:**
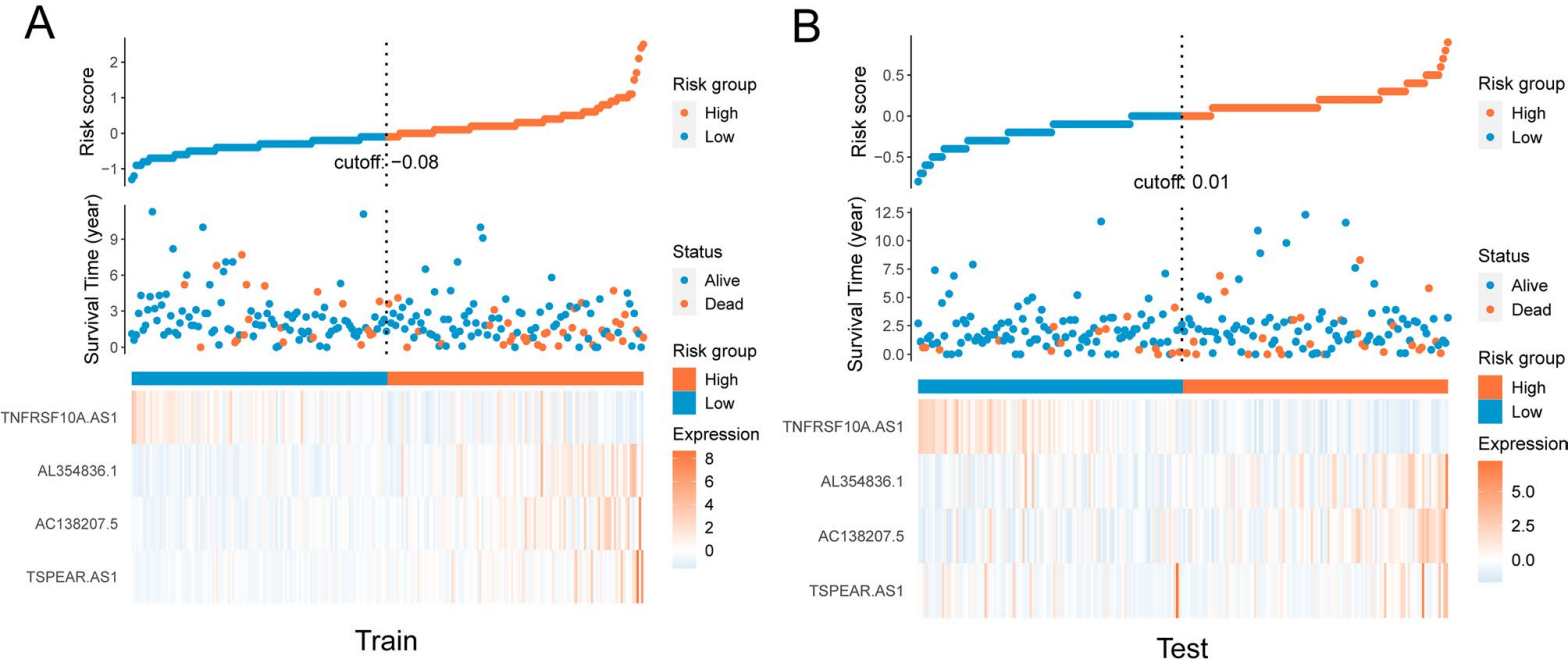
Risk core visualization between the train and test set.

**Figure 6:**
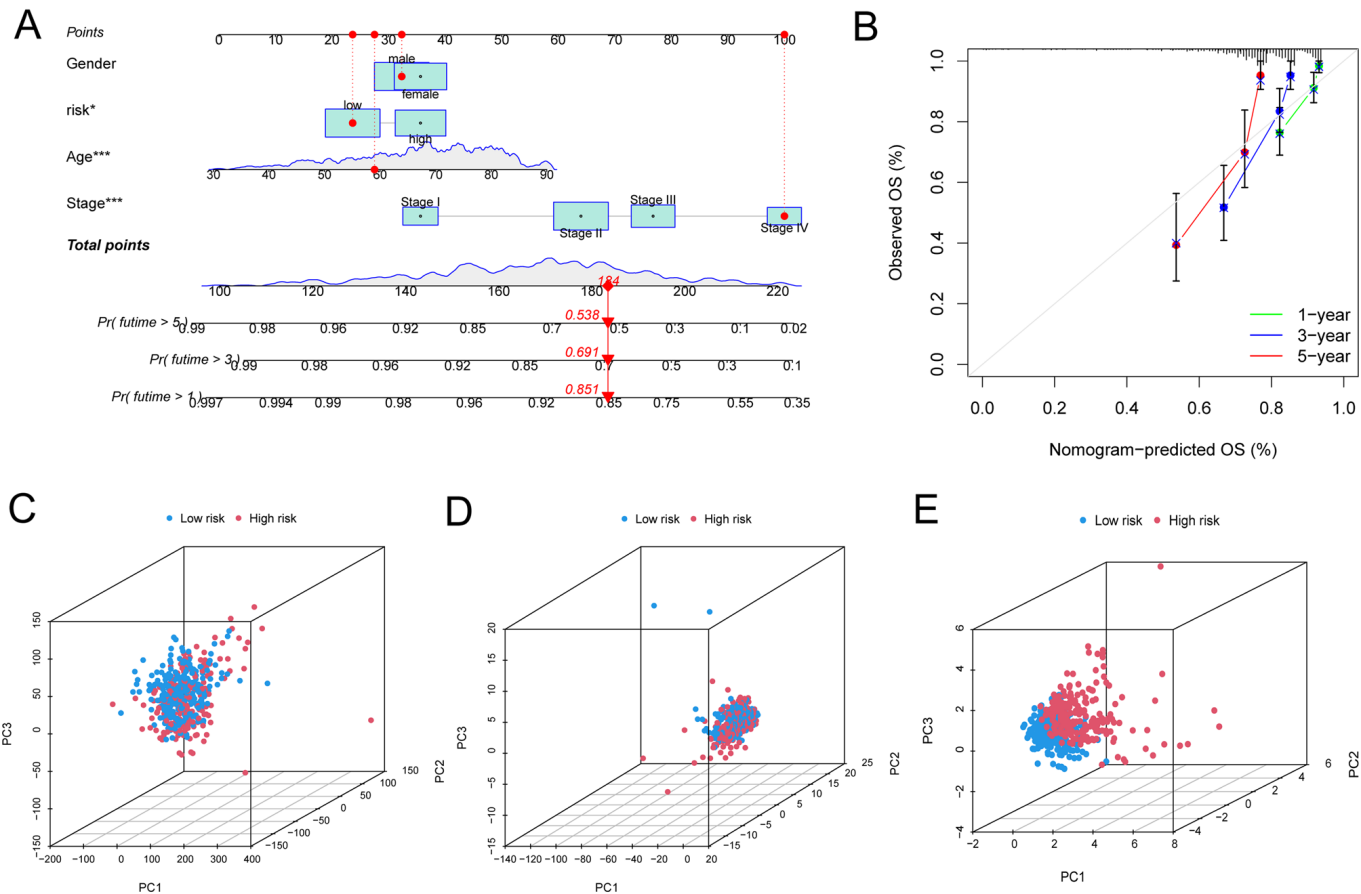
(**A**) Construction and evaluation of a prognostic nomogram. (**B**) The calibration plot of the nomogram predicts the probability of the 1-,3-, and 5-year OS. And principal component analysis between the high- and low-risk groups based on (**C**) entire gene expression profiles, (**D**) 388 LRLs, and (**E**) risk model based on the representation profiles of the 4 prognostic LRLs.

**Figure 7:**
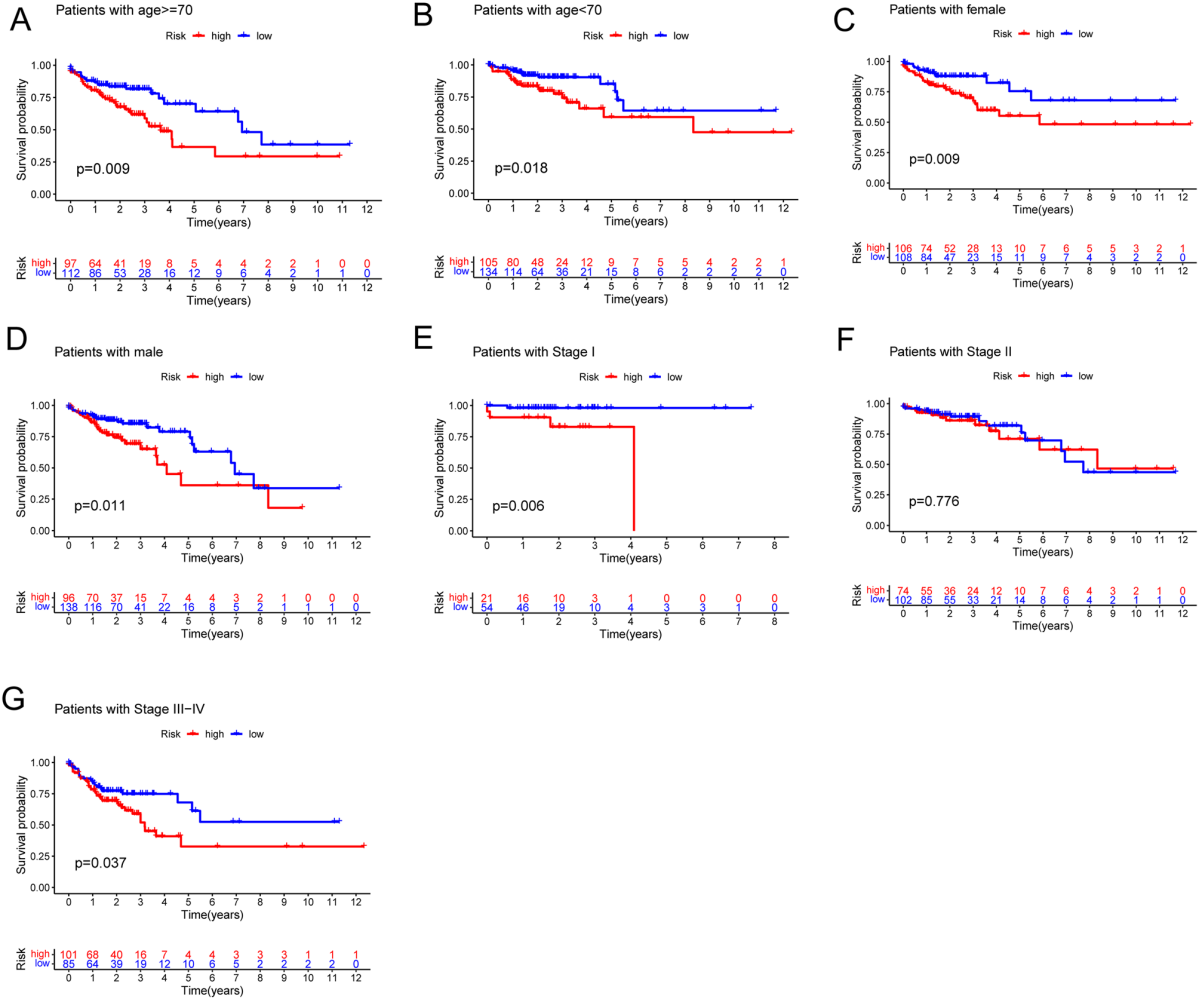
Kaplan–Meier curves of OS differences stratified by gender, age, or TNM stage between the high- and low-risk groups. *p* < 0.05 was considered significant.

The risk score, survival state, and expression of four prognostic LRLs of each colon cancer patient were depicted in Fig. 5A (Train group) and Fig. 5B (Test group), meahwhile, to investigate the reliability of the risk model in colon cancer patients, the prognostic nomogram plot containing the risk score, stage, age and gender was constructed in the study (Fig. 6A), and calibration plot indicated excellent agreement between prediction and actual risk (Fig. 6B). Furthermore, we performed principal component analysis (PCA) to speculate the separation ability of lncRNAs that forms the risk model. Compared with the whole transcriptome expression pattern, lysosome-related lncRNAs, and prognostic LRLs, lncRNA that forms risk model better separates the high-risk group from the low-risk group. (Fig. 6C). Clinical features were separated to scrutinize the universality of the risk score model. As shown, the prediction of OS of the risk score model was accurate in age (Fig. 7A,B), gender (Fig. 7C,D), and stage (Fig. 7E-G). Subsequently, these findings indicated that our risk score model was standard for all colon cancer patients. These results suggested that the risk score is an independent prognostic biomarker for all colon cancer patients.

### Immune microenvironment of the lysosome related lncRNA signature

**Figure 8:**
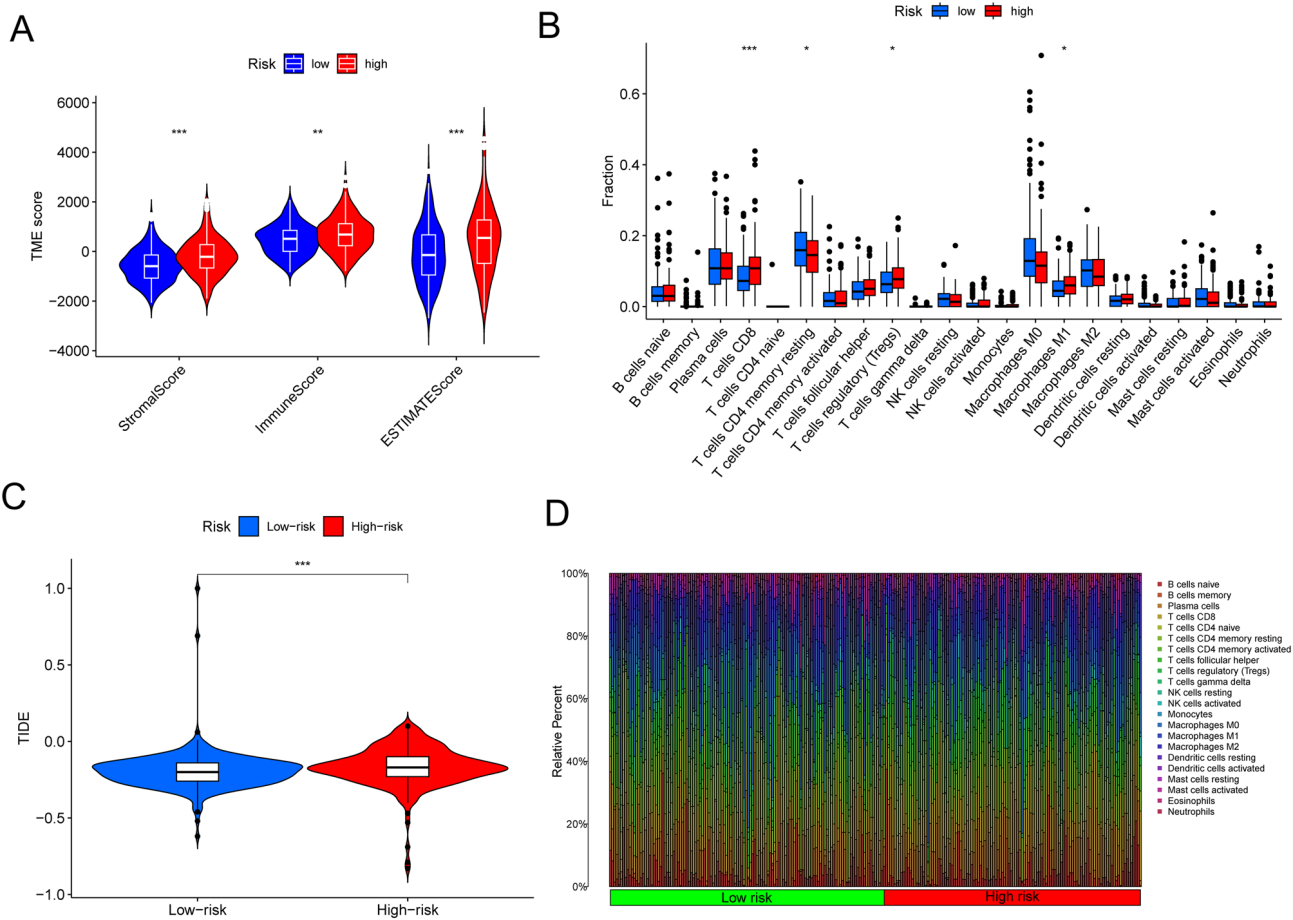
Estimation of tumor immune microenvironment and immune infiltration using the lysosome-related lncRNA model in the TCGA entire set. (**A**) Estimate algorithm between high and low risk group. (**B**) The infiltration of 22 immune cells between high and low risk group using the ssGSEA algorithm. (**C**) Tumor Immune Dysfunction and Exclusion (TIDE) score between high and low risk group. (**D**) Stacked bar chart shows distribution of 22 immune cells in the high- and low-risk groups. *P < 0.05, **P < 0.01, ***P < 0.001.

Tumor microenvironment is a complex component composed of tumor cells, immune cells, blood vessels and surrounding matrix, which could promote or inhibit the occurrence and development of tumors through an interaction mode^36,37^. In this study, we evaluated the association between the risk score and TME by using the estimate algorithm, and found that the high- risk group was obviously more expressed than the low- risk group in the three total score (estimate score, immune score and stromal score) (Fig. 8A). In order to identify the correlation of 4 lysosome-related lncRNA signature with tumor immune response, we assessed the correlation between the risk score and 22 types of tumor infiltrating immune cells (TIICs) in colon cancer and showed that the expression of the CD4 memory resting T cells in the high- risk group was lower than that of the low- risk group. While in the CD8 T cells, Tregs, M1 macrophages, the high- risk group was dominated (Fig. 8B). Furthermore, the tumor immune dysfunction and exclusion (TIDE) score was also examined in this study to further establish the link between the LRL-related signature and immunity, and the result demonstrated that high- risk group has a higher proportion on TIDE score compared with the low- risk group (Fig. 8C). Meanwhile, the distribution difference of 22 TIICs in these two groups was also illustrated in the study (Fig. 8D).

**Figure 9:**
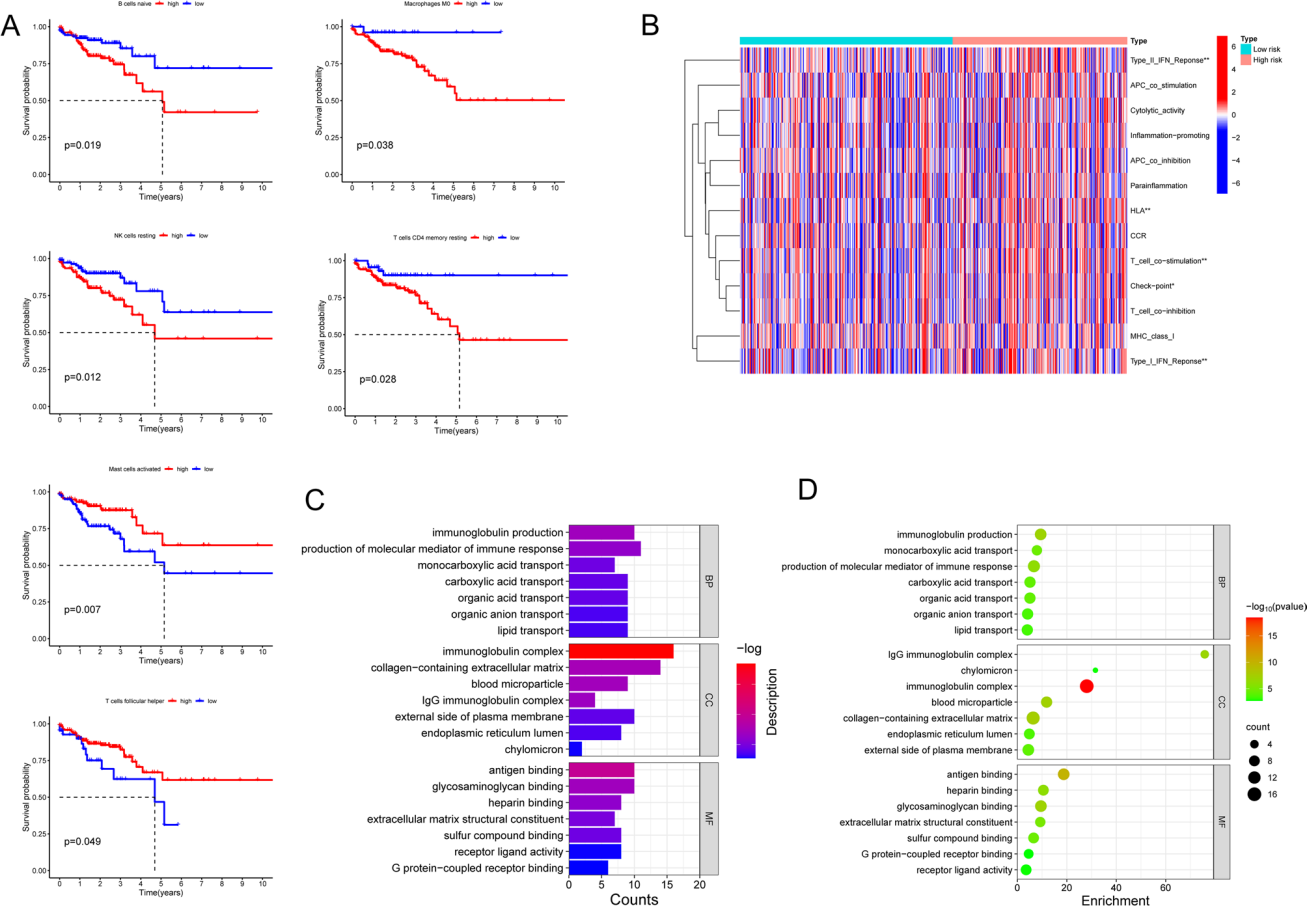
Survival related immune cells (**A**) and immune function enrichment pathways (**B**) analysis between high and low risk group. (C and D) GO enrichment analysis between high- and low- risk group.

Then, we also conducted a survival analysis base on the TIICs and our findings indicated that high level of naive B cell, M0 macrophages, resting NK cells and CD4 resting T cells trend towards a worse OS, while high level of activated mast cell and follicular helper T cell infiltration tend to a longer OS (Fig. 9A), meanwhile, analysis was conducted to unveil the different immune functions between high- and low- risk groups, and the result indicated that the type II IFN response, HLA, T cell co-stimulation, checkpoint and type I IFN response were significantly differed in these two classifications (Fig. 9B). To reveal comprehensive and potential biological functions of the LRL-related signature, we also extracted differential lncRNAs between high- and low- risk groups using Gene Ontology (GO) enrichment analysis (including biological processes (BP), cellular component (CC), and Molecular function (MF)) revealed that the differentially expressed genes between high- and low- risk group were mainly enriched in regulating the immunoglobulin production, production of molecular mediator of immune response, immunoglobulin complex and collagen-containing extracellular matrix, antigen binding, and glycosaminoglycan binding (Fig. 9C,D). Taking the findings into consideration, we concluded that the lysosome-related lncRNA signature could provide new motivate and exert a favourable prediction of immune response of colon patients.

### Association between LRLs_score and TMB, MSI and drug sensitivity

**Figure 10:**
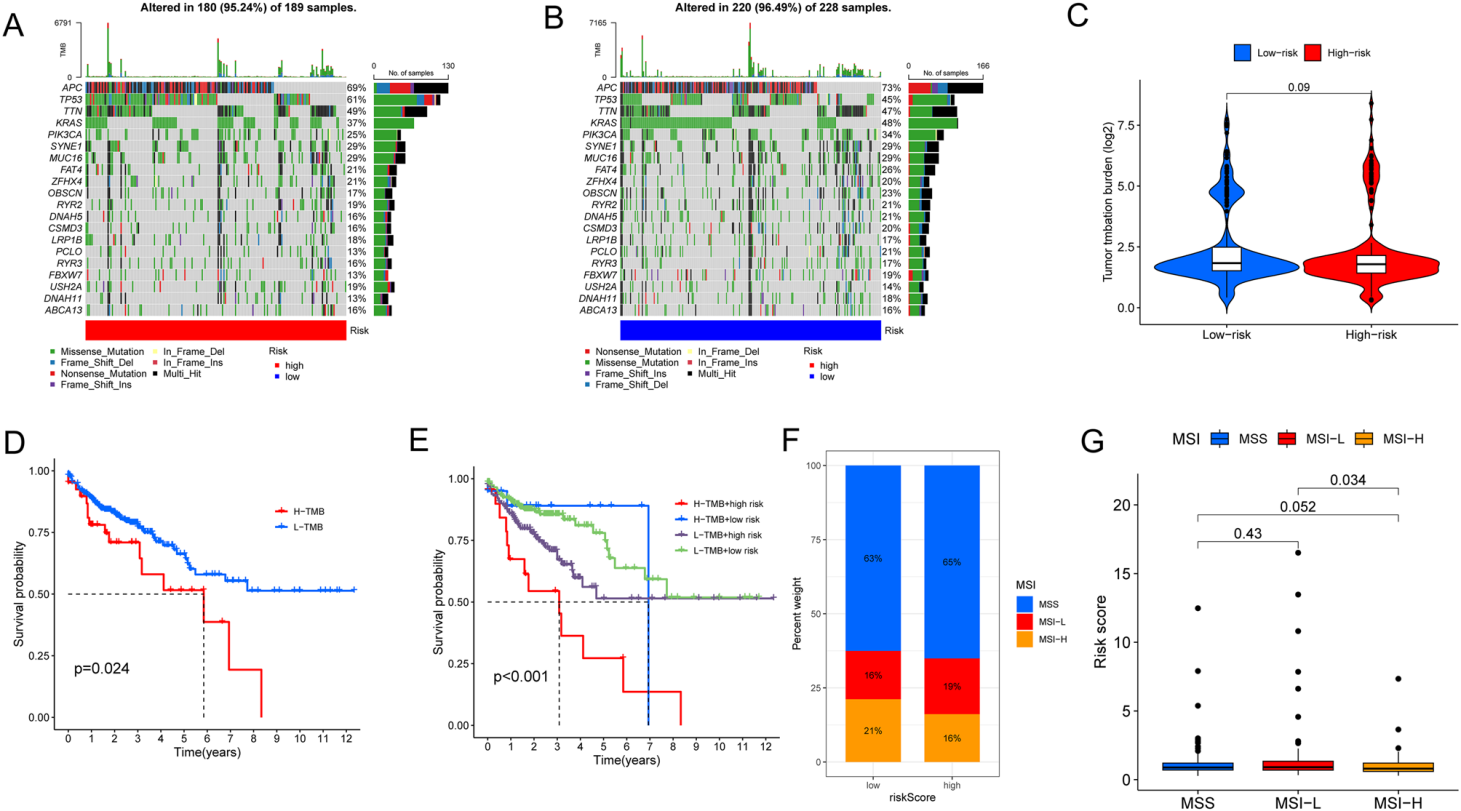
Multifaceted analysis between high and low risk groups. (**A**) Waterfall diagram of mutations between high and low risk groups. (**B**) Relationships between LRLs_score and TMB. (**C**) Survival analysis between high-TMB and low-TMB. (**D**) Survival analysis among H-TMB + high risk, L-TMB + high risk, H-TMB + low risk and L-TMB + low risk. (**E**, **F**) Relationships between LRLs_score and MSI. TMB: tumor mutation burden. MSI: Microsatellite instability. *P* < 0.05 was considered significant.

Tumor mutational burden (TMB) was considered a promising indicator and displayed a strong predictive ability of the clinical outcome after immune checkpoint inhibitors therapy^38,39^. In this study, we analyzed the somatic mutation of colon cancer samples from the TCGA database and found that the top ten mutated genes in colon cancer samples were APC, TP53, TTN, KRAS, PIK3CA, SYNE1, MUC16, FAT4, ZFHX4 and OBSCN. Of which, the mutation rates of APC and KRAS were higher in low- risk group as compared to the high- risk group, however, the mutation rates of TP53 and TTN in low- risk group were completely opposite (Fig. 10A,B). However, the total mutation frequency of these two groups existed no obvious significance (Fig. 10C). Further, we delineated a Kaplan–Meier survival curve to visualize the different clinical outcomes between the high- and low-TMB combined risk score model, and the results showed TMB only affects the survival of patients in the high-risk score group (Fig. 10F,G).

**Figure 11:**
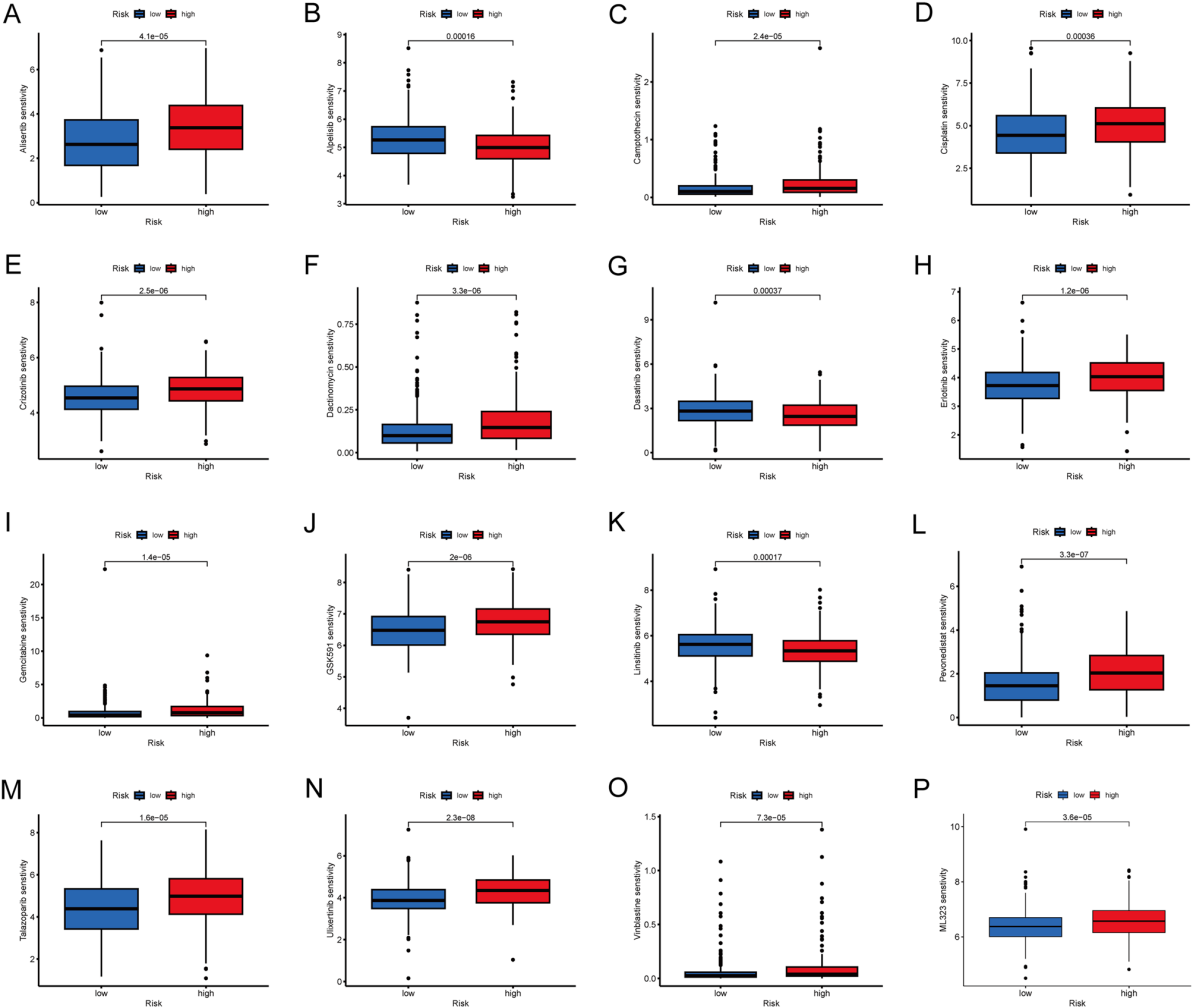
Relationships between LRLs_score and drug sensitivity (**A**–**P**). *P* < 0.05 was considered significant.

Microsatellite instability (MSI) has also become an index to measure immune efficiency and predict immune outcomes in tumor patients^40,41^. There are significant differences between MSI-H and MSI-L, but no differences between MSS and MSI (Fig. 10D). The low-risk group may have a better immunotherapy efficiency since the high-risk group had a higher proportion of MSS while the low-risk group had a higher proportion of MSI-H (Fig. 10E), which might unravel one of the reasons that lead to the worse prognosis of high-risk group patients. Besides, we also performed chemical drug sensitivity analysis in colon cancer, and suggested that the high- risk group respond better to Alisertib, Camptothecin, Cisplatin, Crizotinib, Dactinomycin, Erlotinib, Gemcitabine, GSK591, Pevonedistat, Talazoparib, Ulixertinib, Vinblastine, and ML323, as seen in Fig. 11. However, Alpelisib, Dasatinib and Linsitinib all performed well in the low- risk group. These results suggested the prognostic model may offer effective direction on the immunotherapy and clinical response.

### Validation and assessment of single LRL

We further validated the expression levels and prognostic value of four selected LRLs by investigating their expression levels in both colon cancer cell lines and patients. This was done using qRT-PCR and analyzing clinical data obtained from the TCGA database.

Our findings showed that TNFRSF10A-AS1, TSPEAR-AS1, and AL354836.1 were upregulated in both colon cancer patients and cell lines. On the other hand, AC138207.5 acted as a favorable factor in colon cancer patients (Supplementary Fig. 1A-C, 2A-C, 3A-C, 4A, B). Regarding prognosis, increased levels of TNFRSF10A − AS1 and AL354836.1 were associated with poorer overall survival (Supplementary Fig. 1D, 3D). In addition, elevated levels of TSPEAR-AS1 were associated with poorer disease-specific survival, suggesting that patients with high TSPEAR-AS1 expression were more likely to die from colon cancer rather than other causes (Supplementary Fig. 2E). Interestingly, AC138207.5 exhibits high expression levels in normal samples but has limited prognostic value. We propose that this lncRNA functions as a correction factor, enhancing the accuracy of our risk model (Supplementary Fig. 4A-F).

## Discussion

Lysosomes has been proven to regulate the microenvironment of colon cancer and thereby influencing the prognosis of tumor patients. Previous studies have demonstrated that LYSET, a newly reported lysosomal enzyme transporter, could help tumor cells decompose proteins for nutrition and further accelerate tumor deterioration and proliferation^42,43^. In colon cancer, FUT8 was found to be a therapy target by mediating lysosomal proteolysis^44^. Recently, a study constructed a prognostic model with regard to subtypes of colon cancer based on lysosome-related genes, and found that a higher prognostic LRGs_score was more likely to lead to bad endpoint^45^. However, the potential biological functions of lysosome-related lncRNAs in colon cancer has not been discovered.

In this study, we downloaded RNA-seq, clinical information, and mutation profile of patient samples from TCGA-COAD cohort, to screen the DEGs with the criterion of |log _2_ (fold change) |> 1 and a false discovery rate (FDR) < 0.05. Then, we found 10 lysosome-related DEGs through overlapping the DEGs with lysosome-related genes. Further, we investigated the somatic mutations, CNVs, and prognostic value of these 10 genes. Based on these 10 genes, we established a LRDEGs-lncRNA co-expression network to find the lysosome-related lncRNAs with the criteria of |R|> 0.4 and *p* < 0.05. Three hundred eighty-eight lncRNAs was searched out as lysosome-related lncRNAs for further analysis. We used univariate COX-regression analysis to assess the impact of each lncRNA on OS. Ten lncRNAs were considered as prognostic lncRNA for the establishment of prognostic model. Multivariate COX-regression was employed for the calculation of corresponding coefficient, Lasso analysis was employed to construct the risk model, and machine learning tools to separate the samples into train and test groups. Subsequently, a risk model of four signature LRL was established, including TNFRSF10A-AS1, AL354836.1, AC138207.5, and TSPEAR-AS1. Through merging the clinical data, our findings indicated that the risk model was significantly related to OS and DSS. Besides, we also demonstrated that relationship between the risk model and the clinical features such as age, clinical stage, and gender. These findings indicated the LRL signature of colon cancer mat provide key information on the individualized treatment. The heterogeneity of immune microenvironment is the main reason for the difference of immunotherapy response. Herin, we performed TME score analysis and the result indicated that high- risk group was substantially higher than those of the low- risk group, meanwhile, we also found that a higher TMB in the high- risk group was related to poor prognosis, which suggested that the model may apply for evaluating the efficiency of patients with different extent of TMB. MSI, another essential immunotherapy response indicator, was also applied for the clinical significance of the risk model. The results displayed high-risk group had a higher proportion of MSS while the low-risk group had a higher proportion of MSI-H, which further explain the reason that the high- risk group has shorter survival period. Furthermore, we also detected the relation between the two group and 22 TIICs, and found that CD8 T cell and M1 macrophage occupied a higher proportion in the high- risk group, suggesting that the risk model may be an effective parameter for observing inflammatory response and immune tolerance. Finally, the association between chemotherapeutic drugs and the risk group, and found that cisplatin and pevonedistat were more sensitive in the high- risk group while Alpelisib, Dasatinib, Linsitinib were more sensitivity in the low- risk group, which may help physicians choose more appropriate drugs for colon cancer treatment.

Nowadays, computational biology-based theoretical modeling studies on gene signaling networks have been equally important for the study of understanding regulatory mechanisms and finding potential therapeutic targets in diseases. Especially, ordinary differential equation (ODE)-based modeling and single-cell RNA sequencing focuses more on dynamic variables, simulates and predictes the dynamic behavior in biological systems^46–48^. In this study, we also employed a vaireity of bioinformatics analysis methods and online tools to excavate innovate biomarkers for colon cancer patients, however, the above more sophisticated algorithms have not been used to analyze the lysosomal-related lncRNAs profile in colon cancer. Another limitation of this study is that, our risk model is based on the data from TCGA database with limited clinical sample, further clinical validation is still in need. Besides, lysosome is an organ closely related to metabolism, interestingly, a study has developed a deep learning model as MDA-AENMF to predict the potential associations between metabolites and diseases, which also may detect how these LRLs metabolically stimulate the progression of colon cancer^49^. Previously, it has been confirmed that mRNA can drive protein-mediated phase separation, but the phase separation of lysosome related lncrnas in colon cancer has not been reported^50^. Our future studies will further reveal the role of these LRLs in tumorigenesis and development from the perspective of lncRNA phase separation. In general, from mining biologically significant lysosome-related lncRNAs to targeting potentially valuable proteins, shaping gene-protein interaction network aggregation, such as using deep learning algorithm DMFGAM and graphing convolutional network with graph attention network^51,52^, will be the direction and focus of our future efforts, aiming for exploring promising biomarkers and powerful strategies targeting colon cancer patients.

### Supplementary Information

Supplementary Figure 1.

Supplementary Figure 2.

Supplementary Figure 3.

Supplementary Figure 4.

Supplementary Legends.

Supplementary Table S1.

## Data Availability

The data used in this study are available from the corresponding authors.

## Abbreviations

LncRNAs: Long non-coding RNAs
LRLs: Lysosome-related lncRNA
DEGs: Differentially expressed genes
LRGs: Lysosome related genes
TMB: Tumor mutational burden
MSI: Microsatellite instability
