# Supplementary figures and images for: Development and validation of a novel lysosome-related LncRNA signature for predicting prognosis and the immune landscape features in colon cancer
Source: Sci Rep. 2024 Jan 5;14:622. doi: 10.1038/s41598-023-51126-9 (PMC10770065; doi:10.1038/s41598-023-51126-9)

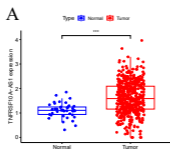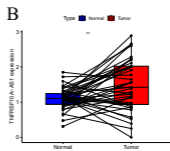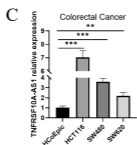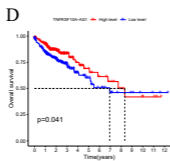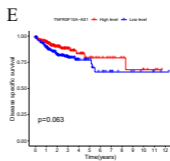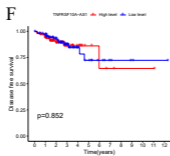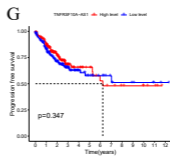

Supplement: Supplementary file 1 — Supplementary Figure 1. [file 41598_2023_51126_MOESM1_ESM.pdf]

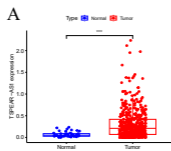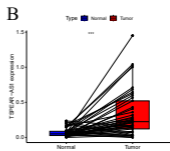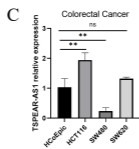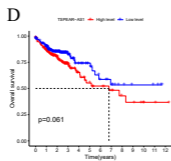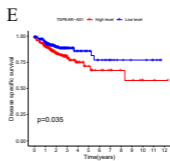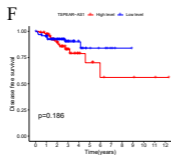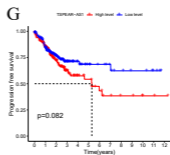

Supplement: Supplementary file 2 — Supplementary Figure 2. [file 41598_2023_51126_MOESM2_ESM.pdf]

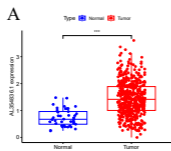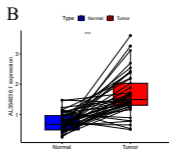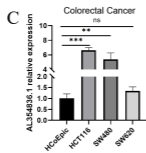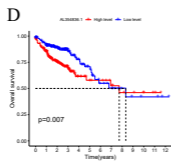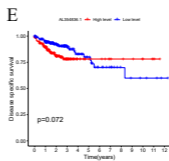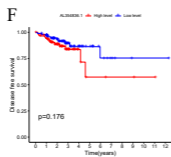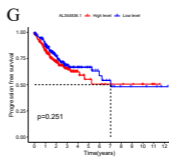

Supplement: Supplementary file 3 — Supplementary Figure 3. [file 41598_2023_51126_MOESM3_ESM.pdf]

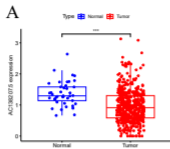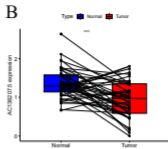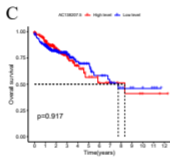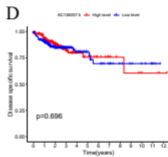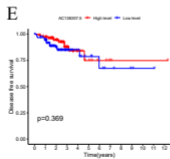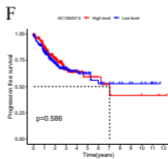

Supplement: Supplementary file 4 — Supplementary Figure 4. [file 41598_2023_51126_MOESM4_ESM.pdf]
