# Supplementary material for: Development and validation of a novel lysosome-related LncRNA signature for predicting prognosis and the immune landscape features in colon cancer
Source: Sci Rep. 2024 Jan 5;14:622. doi: 10.1038/s41598-023-51126-9 (PMC10770065; doi:10.1038/s41598-023-51126-9)
Supplement: Supplementary file 5 — Supplementary Legends. [file 41598_2023_51126_MOESM5_ESM.docx]

**Supplementary figure 1:** Prognostic value and vitro validation of TNFRSF10A-AS1 expression level in colon cancer cell lines. (A) Differential expression of TNFRSF10A-AS1 in normal and colon cancer samples. (B) Forty-one paired differential analysis between normal and cancer samples in TCGA-COAD cohort. (C) The expression of TNFRSF10A-AS1 among normal colonic epithelial cell line (HCoEpic) and colon cancer cell lines (HCT116, SW480, SW620). (D) Overall survival analysis base on the expression level of TNFRSF10A-AS1. (E) Disease-specific survival analysis base on the expression level of TNFRSF10A-AS1. (F) Disease-free survival analysis base on the expression level of TNFRSF10A-AS1. (G) Progression-free survival analysis base on the expression level of TNFRSF10A-AS1.

**Supplementary figure 2:** Prognostic value and vitro validation of TSPEAR−AS1 expression level in colon cancer cell lines. (A) Differential expression of TSPEAR−AS1 in normal and colon cancer samples. (B) Forty-one paired differential analysis between normal and cancer samples in TCGA-COAD cohort. (C) The expression of TSPEAR−AS1 among normal colonic epithelial cell line (HCoEpic) and colon cancer cell lines (HCT116, SW480, SW620). (D) Overall survival analysis base on the expression level of TSPEAR−AS1. (E) Disease-specific survival analysis base on the expression level of TSPEAR−AS1. (F) Disease-free survival analysis base on the expression level of TSPEAR−AS1. (G) Progression-free survival analysis base on the expression level of TSPEAR−AS1.

**Supplementary figure 3:** Prognostic value and vitro validation of AL354836.1 expression level in colon cancer cell lines. (A) Differential expression of AL354836.1 in normal and colon cancer samples (p<0.001). (B) Forty-one paired differential analysis between normal and cancer samples in TCGA-COAD cohort. (C) The expression of AL354836.1 among normal colonic epithelial cell line (HCoEpic) and colon cancer cell lines (HCT116, SW480, SW620). (D) Overall survival analysis base on the expression level of AL354836.1. (E) Disease-specific survival analysis base on the expression level of AL354836.1. (F) Disease-free survival analysis base on the expression level of AL354836.1. (G) Progression-free survival analysis base on the expression level of AL354836.1.

**Supplementary figure 4:** Prognostic value and vitro validation of AC138207.5 expression level in colon cancer cell lines. (A) Differential expression of AC138207.5 in normal and colon cancer samples (p<0.001). (B) Forty-one paired differential analysis between normal and cancer samples in TCGA-COAD cohort. (C) Overall survival analysis base on the expression level of AC138207.5. (D) Disease-specific survival analysis base on the expression level of AC138207.5. (E) Disease-free survival analysis base on the expression level of AC138207.5. (F) Progression-free survival analysis base on the expression level of AC138207.5.
