# Supplementary material for: Development and validation of a novel lysosome-related LncRNA signature for predicting prognosis and the immune landscape features in colon cancer
Source: Sci Rep. 2024 Jan 5;14:622. doi: 10.1038/s41598-023-51126-9 (PMC10770065; doi:10.1038/s41598-023-51126-9)
Supplement: Supplementary file 6 — Supplementary Table S1. [file 41598_2023_51126_MOESM6_ESM.docx]

Table S1:

Quantitative Real-Time PCR primers used in the study

| **Name** | **Sequences of Primer** |
| --- | --- |
| TNFRSF10A-AS1 Forward Primer | TCTCAGATCACGTGACCTTGA |
| TNFRSF10A-AS1 Reverse Primer | GTGGGCAGCTCTCATCCTAA |
| TSPEAR-AS1 Forward Primer | CCCACCCTGATCCCTCTAGT |
| TSPEAR-AS1 Reverse Primer | ATGGAACCACCGGAACCAAG |
| AL354836.1 Forward Primer | CTCCCCAGCACAGCTTCTAC |
| AL354836.1 Reverse Primer | TGCAGCACCATCAAAAACGGT |
| β-actin Forward Primer | TGGCACCCAGCACAATGAA |
| β-actin Reverse Primer | CTAAGTCATAGTCCGCCTAGAAGCA |
